# Neurons That Underlie *Drosophila melanogaster* Reproductive Behaviors: Detection of a Large Male-Bias in Gene Expression in *fruitless*-Expressing Neurons

**DOI:** 10.1534/g3.115.019265

**Published:** 2016-05-31

**Authors:** Nicole R. Newell, Felicia N. New, Justin E. Dalton, Lauren M. McIntyre, Michelle N. Arbeitman

**Affiliations:** * Biomedical Sciences Department and Center for Genomics and Personalized Medicine, Florida State University, College of Medicine, Tallahassee, Florida 32306; † Genetics Institute and Department of Molecular Genetics and Microbiology, University of Florida, Gainesville, Florida 32610-0266

**Keywords:** sex differences, courtship behaviors, *Drosophila*, sex hierarchy, *fruitless*, genetics of sex

## Abstract

Male and female reproductive behaviors in *Drosophila melanogaster* are vastly different, but neurons that express sex-specifically spliced *fruitless* transcripts (*fru P1*) underlie these behaviors in both sexes. How this set of neurons can generate such different behaviors between the two sexes is an unresolved question. A particular challenge is that *fru P1*-expressing neurons comprise only 2–5% of the adult nervous system, and so studies of adult head tissue or whole brain may not reveal crucial differences. Translating Ribosome Affinity Purification (TRAP) identifies the actively translated pool of mRNAs from *fru P1*-expressing neurons, allowing a sensitive, cell-type-specific assay. We find four times more male-biased than female-biased genes in TRAP mRNAs from *fru P1*-expressing neurons. This suggests a potential mechanism to generate dimorphism in behavior. The male-biased genes may direct male behaviors by establishing cell fate in a similar context of gene expression observed in females. These results suggest a possible global mechanism for how distinct behaviors can arise from a shared set of neurons.

Adult reproductive behaviors in *Drosophila melanogaster* are directed predominately by a set of neurons that express sex-specifically spliced *fruitless* transcripts (*fru P1* transcripts) (Ito *et al.* 1996; Ryner *et al.* 1996; Demir and Dickson 2005; Manoli *et al.* 2005; Stockinger *et al.* 2005; Dickson 2008; and reviewed in Siwicki and Kravitz 2009). These neurons are largely detected as dispersed clusters, comprising ∼2–5% of neurons in the central and peripheral nervous system, of both males and females (Lee *et al.* 2000; Manoli *et al.* 2005; Stockinger *et al.* 2005). The positions and number of *fru P1*-expressing neurons are notably similar in males and females, though there are evident differences in neuronal arborization volume, neuron number, and physiology (Kimura *et al.* 2005; Manoli *et al.* 2005; Stockinger *et al.* 2005; Datta *et al.* 2008; Cachero *et al.* 2010; Yu *et al.* 2010; Ito *et al.* 2012; Kohl *et al.* 2013; and reviewed in Yamamoto and Koganezawa 2013).

How sex-specific differences arise in a set of neurons with a shared developmental trajectory and genome to ultimately direct the distinct adult behaviors is not understood. Additionally, in terms of gene expression, it is not known what is common to both male and female *fru P1*-expressing neurons, and whether that is distinct from other neurons. To address these questions, we leverage a molecular-genetic tool that allows enrichment of mRNAs resident in ribosomes (Thomas *et al.* 2012), in a cell-type-specific manner, to discover the characteristics of the translatome of male and female *fru P1*-expressing neurons.

In *Drosophila*, adult reproductive behaviors are regulated downstream of the sex hierarchy, an alternative pre-mRNA splicing cascade, responsive to the number of the X chromosomes, that directs the production of sex-specific transcription factors encoded by *fruitless* (*fru*) and *doublesex* (*dsx*) (see Figure 1) (reviewed in Yamamoto 2007; Villella and Hall 2008). *fru* is a complex locus that has multiple promoters, with the *fru P1* promoter directing production of the transcripts that are sex-specifically alternatively spliced (Ito *et al.* 1996; Ryner *et al.* 1996). This splicing results in production of male-specific isoforms (Fru^M^) (reviewed in Manoli *et al.* 2006; Dauwalder 2011). There is an early stop codon in the female-specific *fru P1* transcript, and so there is no functional product predicted (Usui-Aoki *et al.* 2000). *dsx* directs sex differences in body morphology, and also plays a role in the nervous system to specify the potential for reproductive behaviors, with some overlap in expression with *fru P1* (Lee *et al.* 2002; Rideout *et al.* 2007; Kimura *et al.* 2008; Sanders and Arbeitman 2008; Rideout *et al.* 2010; Robinett *et al.* 2010; and reviewed in Yamamoto and Koganezawa 2013).

**Figure 1:**
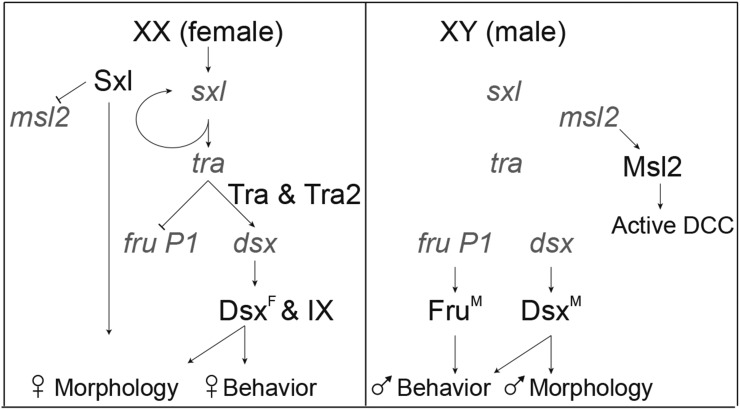
*Drosophila* sex determination hierarchy. In *Drosophila* differences in somatic tissues are directed by the sex determination hierarchy. In response to the number of X chromosomes, alternative pre-mRNA splicing occurs at the top of the hierarchy (*sxl*, *tra* and *tra-2* are splicing factors that direct alternative pre-mRNA splicing of *dsx* and *fru P1*), leading to sex-specific production of Dsx and Fru transcription factors. In females, production of Sxl also inhibits translation of *msl2*, resulting in the absence of dosage compensation in females, as the dosage compensation complex (DCC) is not formed in the absence of MSL2. In females, Dsx^F^ together with Ix regulate gene expression to direct female-specific behavior, morphology, and physiology. In males, Dsx^F^ and Fru^M^ regulate gene expression to direct male-specific behavior, morphology, and physiology.

Several studies have demonstrated that the neurons that express *fru P1* in males are important for the elaborate male courtship display (Ito *et al.* 1996; Ryner *et al.* 1996; Villella *et al.* 1997; Anand *et al.* 2001; Lee *et al.* 2001; Demir and Dickson 2005; Manoli *et al.* 2005; von Philipsborn *et al.* 2011; reviewed in Yamamoto and Koganezawa 2013; Tran *et al.* 2014; Zhou *et al.* 2015). *fru P1*-expressing neurons also direct female reproductive behaviors, including receptivity to mating, egg laying, food preference after mating, and rejection behaviors (Kvitsiani and Dickson 2006; Yapici *et al.* 2008; Hasemeyer *et al.* 2009; Yang *et al.* 2009). How *fru P1*-expressing neurons drive both male and female adult behavior is not understood. We hypothesize that these neurons translate a different set of proteins in males and females. Our study focuses on the translatome, as this is currently one of the only ways to assess the differences in the protein production specifically in *fru P1*-expressing neurons.

Here, we identify mRNAs that are enriched in the translatome of male and female *fru P1*-expressing neurons in 16- to 24-hr adults, using the Translating Ribosome Affinity Purification (TRAP) method (for review and discussion of cell-specific tools see Tallafuss *et al.* 2014). We drive expression of the GFP-tagged variant of RpL10A in *fru P1*-expressing neurons, using Gal4 driven by the *fru P1* promoter (Manoli *et al.* 2005; Thomas *et al.* 2012). We analyze five independent biological replicates to increase our power to detect significant differences in gene expression (Liu *et al.* 2014). We identify sex-differences in the translatome of *fru P1*-expressing neurons, with the majority of genes showing male-biased transcript abundance. We also identify genes with products that are present in the ribosome of both sexes, which we find are distinct from those that are enriched in all neurons of the adult head, also identified using the TRAP approach (Thomas *et al.* 2012). This may be because *fru P1*-expressing neurons are a small subset of all neurons. The results point to the importance of understanding the translatome in a cell-type-specific manner. An immediate question suggested by these findings is how much of this male-biased TRAP mRNA is a result of the Fru^M^ protein itself, as this protein is made only in males. To address this, we compare the results of this study to our previous analyses aimed at identifying targets of Fru^M^ (Dalton *et al.* 2013).

## Materials and Methods

### Flies

Flies were raised on standard cornmeal food medium at 25° on a 12-hr light and 12-hr dark cycle. Flies that express the GFP-tagged variant of RpL10A in *fru P1*-expressing neurons are the genotypes: *w/(w or* Y); *P[w^+mC^*, *UAS-Gal4]/P[w^+mC^*, *UAS-GFP*::*RpL10A]*; *fru P1-Gal4/+* (Manoli *et al.* 2005; Thomas *et al.* 2012). We note that this *fru P1-Gal4* allele has been shown to masculinize the female nervous system when homozygous, but in this study it is heterozygous (Ferri *et al.* 2008).

### Tissue collection

Libraries were prepared from five independent biological replicates for each condition. For each replicate, approximately 1000 flies that were 16- to 24-hr posteclosion were used. All flies were collected 0–8 hr posteclosion under anesthetization, and allowed to recover for 16 hr before being snap frozen in liquid nitrogen between ZT1 and ZT2. Snap-frozen whole animals were stored at –80° until heads were collected. Frozen adult heads were separated from bodies by vigorous vortexing and collected using chilled mesh sieves #25 and #40 to separate out the bodies and heads, respectively.

### Polysome immunoprecipitation

Translated mRNA purification was performed as previously described (Doyle *et al.* 2008; Heiman *et al.* 2008; Thomas *et al.* 2012; Heiman *et al.* 2014), with the following modifications: approximately 1000 heads per replicate were collected and homogenized in lysis buffer [20 mM HEPES, pH 7.4, 150 mM KCl, 5 mM MgCl_2_, 0.5 mM DTT, 100 μg/ml Cyclohexamide, 100 U/ml RNaseOUT (Invitrogen), 1 × Complete Protease Inhibitor (Roche)]. The sample was centrifuged at 4° for 10 min at 2000 × *g* and the supernatant was collected. 1,2-diheptanoyl-*sn*-glycero-3-phosphocholine (DHPC; Avanti Polar Lipids, Inc.) and NP-40 were added to a final concentration of 30 mM and 1%, respectively, to the supernatant and incubated on ice for 5 min. The lysate was then centrifuged at 4° for 10 min at 20,000 × *g*; 40 μl of the supernatant was retained and placed directly into 200 μl of Lysis Solution [Ambion MicroPoly(A) Purist Kit] to be used to analyze the input RNA fraction. The remaining supernatant was added to Dynabeads Protein G magnetic beads (Novex) conjugated to mouse anti-GFP (Memorial Sloan-Kettering Monoclonal Antibody Facility19C8/19F7). The lysate-bead slurry was incubated for 30 min at 4° followed by washing in Wash Buffer (20 mM HEPES, pH 7.4, 5 mM MgCl_2_, 350 mM KCl, 1% NP-40, 0.5 mM DTT, and 100 μg/ml Cyclohexamide) at 4°. The IP was resuspended in Lysis Solution [Ambion MicroPoly(A) Purist Kit] and mRNA was directly isolated following the manufacturer’s protocol.

### Illumina sequencing library preparation

Poly(A)^+^ transcripts were isolated using Ambion MicroPoly(A)Purist Kit. mRNA was chemically fragmented to a range of approximately 200–500 bp using the Ambion RNA Fragmentation Reagent, and the RNA was purified using Zymo Research RNA Clean & Concentrator-5. First strand cDNA was synthesized using SuperScript II Reverse Transcriptase (Invitrogen) and a combination of 3 µg random hexamers and 0.15 µg oligo(dT)_20_ primers. Following first strand synthesis, the second strand of the cDNA was synthesized by addition of DNA polymerase I (Invitrogen), RNase H (New England Biolabs Inc.), dNTPs and second strand buffer (Invitrogen). The DNA from this and all subsequent reactions was purified using Zymo Research DNA Clean & Concentrator-5 kit. Double-stranded cDNA templates were blunt ended using End-It Repair Kit (Epicentre). Next, A-overhangs were added to both ends with Klenow fragment (3′→5′ exo-minus) (New England Biolabs Inc.). Illumina sequencing adapters were then ligated to both ends of the cDNA templates using Fast-Link DNA Ligation Kit (Epicentre). cDNA templates were amplified by performing polymerase chain reaction (PCR; 18 cycles), which extended the adapter and incorporated a different six base pair index into each sample. The product was isolated by gel purification of 250–550 bp fragments. Samples were pooled and sequenced on the Illumina HiSeq 2500 platform with 100-bp single end reads, and the reads were matched to their corresponding sample via the index.

### Illumina read alignment

Distinct reads (nonduplicate) were aligned to FB5.51 (FlyBase Version 5.51) using Bowtie (Langmead *et al.* 2009) (Version 1.9; -m1 -v3) and LAST (Frith *et al.* 2010) (Version 247; -l 25). Coverage was calculated as Reads Per Kilobase per Million, RPKM (Mortazavi *et al.* 2008), which adjusts for number of reads mapped and region length. Exonic regions with at least one read present for at least half of the replicates for each treatment were analyzed further. Exonic regions with no coverage in all replicates, and those with no coverage in half or more of the replicates for one treatment condition were removed from the analysis. The natural log of the RPKM was taken.

### Differential gene expression

In this study, differential abundance was determined for each exon separately and then existing gene models were used to make determinations about differential abundance of transcript isoforms, as we have previously done (Dalton *et al.* 2013). We report that a gene is differentially expressed if at least one exon from the gene has significant differential expression in the comparison.

Differential gene expression was tested using a linear model where *Y*_ij_ = μ + *t*_i_ + ε_ij_ where *Y* is the log transformed RPKM for the *i*th condition (*i* = male input (MI), Female input (FI), male TRAP (MT), female TRAP (FT)) and jth replicate (*j* = 1,...,5) the residuals were modeled as ei ∼N(0,σ^2^_i_) (Law *et al.* 2014). This accounts for heteroscedasticity (unequal variance) of variances. While not commonly addressed in most RNA-seq studies, this issue is underappreciated and worth serious consideration (Law *et al.* 2014). In these types of experiments, such differences in variances are not unexpected. After the model was determined to fit well for most exons, the following contrasts were performed: μMT = μMI; μFT = μFI; μMT – μMI = μFT – μFI; μMT = μFT; μMI – μFI. All of the contrasts were considered simultaneously in a false discovery rate (FDR) correction (Benjamini and Hochberg 1995). An FDR p-value of ≤ 0.2 was considered significant. The FDR is the proportion of rejections that are expected to be false positives. As with the family-wise error rate, decreasing the FDR results in increasing the FNR (false nondiscovery rate). Due to concerns about type II error, a more liberal FDR was used. The results are similar at lower levels of the FDR, and the full results table of all tests performed is provided with raw and adjusted p-values (Supplemental Material, Table S1 and Table S2).

### Motif analysis

MAST (Bailey and Gribskov 1998) uses a file of motifs (Fru^A, B, and C^ DNA binding motifs determined using SELEX), and a sequence FASTA file to identify Fru binding sites in a region that includes the gene of interest, 2 kb upstream of the transcription start site, and 2 kb downstream of the 3′UTR. All analyses were conducted in SAS.

### Enrichment analyses

Targeted comparisons were made using a Fisher’s exact test (Beissbarth and Speed 2004). We tested whether there is a significant association of the Fru^M^ regulated genes, as previously determined (Dalton *et al.* 2013), with the male-biased genes identified here. We also tested whether genes regulated by Fru^M^ and male-biased were significantly enriched on a chromosome. A Fisher’s exact test was used to test for enrichment of the Fru binding sites for the A, B, and C DNA binding domains.

Gene Ontology (GO) enrichment analysis was performed using the Gene Ontology Enrichment portal in Flymine v42.1 (Lyne *et al.* 2007). Target list containing FBgns of genes in each list were supplied against the default background list. GO terms, associated p-values, and associated genes for biological processes, cellular components, and molecular functions with a Benjamini-Hochberg corrected p-value cut-off of < 0.05 were compiled for each list. Enriched protein domain analysis was also implemented with the Benjamini-Hochberg correction in the Flymine portal with a p-value cut-off of < 0.05 (Lyne *et al.* 2007). Tissue analyses to identify *Drosophila* tissues with the largest number of genes with significantly high expression were done by comparison to the FlyAtlas data set (Chintapalli *et al.* 2007; Robinson *et al.* 2013).

### Data availability

The accession number for the data is: GSE67743 at the Gene Expression Omnibus.

## Results

This study identifies the transcripts that are actively translated in *fru P1* neurons of males and females, with the goal of providing insight into the complement of protein differences between the sexes in neurons that control sex-specific behaviors. Translating ribosome affinity purification (TRAP) was used to enrich for transcripts from the actively translated mRNA pool in *fru P1*-expressing neurons, from 16- to 24-hr adult male and female head tissues. RNA-sequencing (RNA-seq) was performed on five independent biological replicates, to identify transcripts with differences in abundance, on a genome-wide level, with exon-specific resolution (see Figure S1 for exon-level mapping, and Graze *et al.* 2012).

### Overview of the genes detected in input and TRAP samples

**Figure 2:**
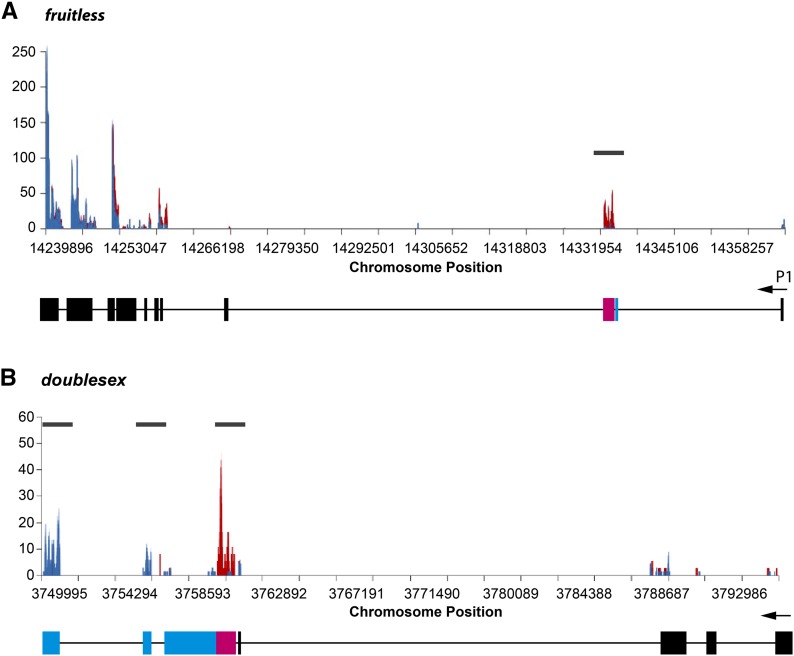
TRAP sequencing read coverage of *dsx* and *fru*. Average normalized read coverage (Y axis) across the (A) *fru* and (B) *dsx* loci from male (blue) and female (red) TRAP samples aligned to the gene structure. For both *fru* and *dsx*, the 5′ end of the transcript is on the right. The gray bar above the count data indicates where there are sex-differences in retention of an exon, because of sex hierarchy regulated alternative pre-mRNA splicing. *fru* has at least 15 different transcripts, and *dsx* has at least six different transcripts (Attrill *et al.* 2016).

We determined that the TRAP technique is working effectively in *fru P1*-expressing neurons. First, we observe that *fru* transcripts are enriched in male and female TRAP samples relative to the input, as we would expect, given that we are using *fru P1-Gal4* to drive expression of the tagged ribosome subunit for purification. However, *fru* also encodes transcripts expressed outside of the *fru P1*-expressing domain, and all *fru* transcripts share common exons (Ryner *et al.* 1996). Thus, we do not expect all *fru* exons to be enriched in the TRAP samples. Four *fru* exons are 1.5-fold higher in male TRAP samples *vs.* male total RNA input, with two statistically significantly higher. One exon is 1.5-fold higher in female TRAP samples *vs.* female RNA input and is statistically significantly higher. The exon that is sex-specifically spliced under sex-hierarchy control is statistically significantly higher in the female TRAP samples compared to the male TRAP samples, as expected given the length difference (see Figure S1 and Figure 2). These results underscore the importance of examining expression at the exon level.

Additionally, though *fru P1* and *dsx* are among genes with relatively low mRNA abundance in the adult head (Chang *et al.* 2011), and are not always consistently detected in gene expression studies (Fear *et al.* 2015), we detect the transcripts, as well as sex-specific differences in splicing (Figure 2), demonstrating the enhanced sensitivity of the technique. We define a detected gene as > 50% of the replicates having at least one exon with at least one sequence read. In this study, 12,442 genes had sequence read coverage, with 12,439 genes detected in the whole head input RNA and 9030 genes detected in the TRAP samples (Table S3). There are 3412 genes that are detected only in the input (Table S3).

Of the 9030 genes detected in the TRAP samples, 8700 are detected in the male TRAP samples and 8297 are detected in the female TRAP samples (Table S3). A similar number of genes was detected in *fru P1* TRAP samples, as detected in the entire head in previous studies using Illumina sequencing (9473, Chang *et al.* 2011) and microarrays (7411, King *et al.* 2012). The TRAP samples have fewer genes detected than the input samples in this study, which is expected, as we are enriching for transcripts from genes expressed in *fru P1*-expressing neurons, whereas whole head samples include other tissue-types, including fat body and muscle. The increase in genes detected overall is likely due to a combination of the increased number of replicates and improvement in technology leading to higher sequencing depth. Thus, TRAP is clearly effective here, and can be used to understand the properties of the *fru P1* translatome.

### Gene expression differences between male and female adult head tissues

**Figure 3:**
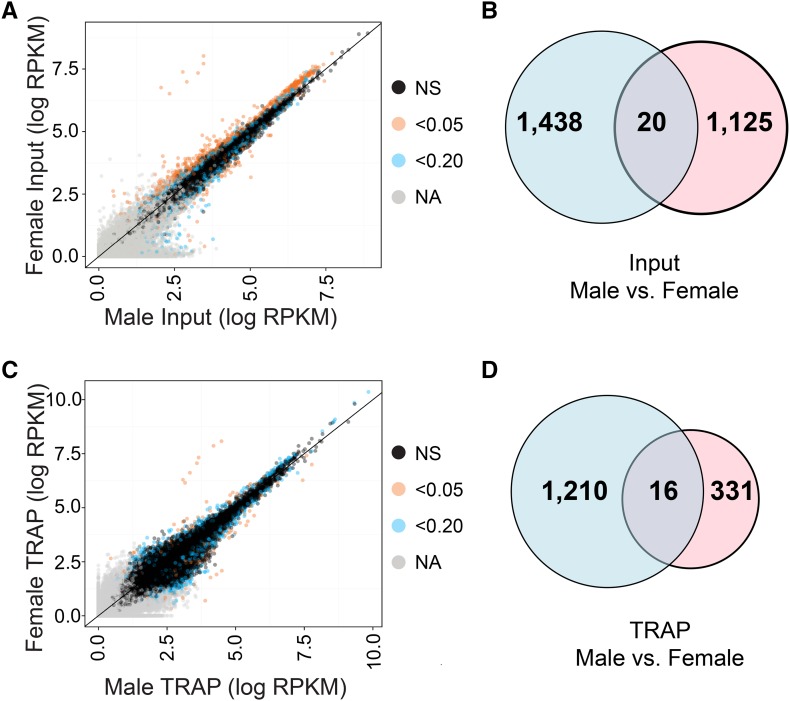
Gene and exon expression differences between males and females in input and TRAP samples. (A) Comparison of log mean RPKM values of male and female input mRNA samples. (B) Comparison of genes with male-biased (blue) and female-biased (pink) expression in input mRNA samples. (C) Comparison of log mean RPKM values of male and female TRAP samples. (D) Comparison of genes with male-biased (blue) and female-biased (pink) TRAP enrichment. A gene can be both male-biased and female-biased if different exons for that gene showed male-biased or female-biased expression. In (A) and (C), exons that were not statistically tested (gray, NA), exons that were not significant (black, NS), exons that were significantly different at an FDR value of < 0.20 (blue), and exons that were significantly different at an FDR value of < 0.05 (orange), are indicated.

We wanted to determine how many genes have sex-specific or sex-biased expression in the input RNA, using exon-level analysis, in contrast to our previous studies where we report sex-differences in expression levels in adult head tissues using gene-level analyses (Goldman and Arbeitman 2007; Chang *et al.* 2011). In this study, 1537 genes have exons that are detected only in input from males, and 294 genes have exons that are detected only in input from females (Table S3). Based on statistical comparisons, we find 1438 (male-biased) and 1125 (female-biased) genes have significant, sex-biased expression in the input mRNA (Figure 3, Table 1, and Table S4). Previously our gene-level, RNA-seq analyses found 815 (male) and 566 (female) genes with significant, sex-biased expression (Chang *et al.* 2011). If we examine the overlap between the two studies, there are 250 male-biased and 214 female-biased genes identified in both studies (Table S4). This difference is not unexpected; the two studies differ in strain background, which has a large impact on sex-biased expression (Catalan *et al.* 2012; Arbeitman *et al.* 2016). We also note that detecting overlap between the two studies is sensitive to our ability to statistically detect expression differences. The genes that are sex-biased in both studies include those with an enrichment of genes with functions in the synapse (male-biased), ribosome and mitotic spindle (female-biased), based on GO analyses (Table S5). There are 20 genes with evidence for sex-specific exon usage (Table 1, Figure 3, and Table S4). This is evidence for alternative transcript isoform usage in males and females—a result consistent with previous studies of the whole body (McIntyre *et al.* 2006) and the head (Telonis-Scott *et al.* 2009; Graze *et al.* 2012).

**Table 1:** Differences between male and female input mRNA

Description	Exons	Genes	GO: Biological Process*^a^*	GO: Cellular Component*^a^*	GO: Molecular Function*^a^*	Protein Domain*^a^*	Tissue Expression (# of Genes)*^b^*
Male-biased in input mRNA	1956	1438	Synaptic transmission	Synapse	Cytoskeletal binding protein	SH3 domain	Brain (881)
			Signaling	Presynapse		PDZ domain	Larval CNS (872)
			Regulation of neurotransmitter levels			Voltage-dependent channel	Thor. Gang (843)
Female-biased in input mRNA	1710	1125	Mitotic spindle elongation	Ribosomal subunit	Structural constituent of ribosome	EGF-like domain	Adult carcass (603)
			Ribosome biogenesis				Adult fat body (563)
							Mated Spermathecae (556)
Sex-specific exon usage		20					

a Gene Ontology (GO) terms and protein domain enrichments were among the most significant for each list.

b The three fly tissues that had the largest number of genes with significantly high expression in the FlyAtlas data set.

### Genes with fru P1 translatome mRNA enrichment relative to input

Next, we wanted to identify the genes that are different between the male and female TRAP samples relative to the input RNA from the same sex. It should be noted that genes expressed in *fru P1*-expressing neurons are likely to be expressed in other cells/neurons of the head, so we do not expect all genes expressed in *fru P1*-expressing neurons to be enriched in the TRAP samples relative to the input (for example see Dalton *et al.* 2009). In this analysis, the null hypothesis is that there is no difference between input and TRAP samples. Further, for an exon to be declared significantly enriched in the TRAP samples, the abundance needed to be higher in the TRAP sample compared to input from the same sex.

We identified 772 genes that are higher in male TRAP samples compared to the male input and 408 genes that are higher in the female TRAP samples compared to the female input (Table 2 and Table S6). These genes are enriched with those that encode neuropeptide receptors, signaling molecule and neurotransmitter secretion products (Table 2). Of these, 476 genes had higher expression in male TRAP only; 112 genes had higher expression in female TRAP only; and 296 genes have higher expression in TRAP samples in both sexes (Table S6). There are 11 genes with evidence for sex-specific exon enrichment in TRAP relative to input mRNA (Table S6).

**Table 2:** *fru P1* TRAP mRNA enrichment relative to input mRNA

Description	Exons	Genes	GO: Biological Process***^a^***	GO: Cellular Component***^a^***	GO: Molecular Function***^a^***	Protein Domain***^a^***	Tissue Expression (# of Genes)*^b^*
Enriched in male TRAP relative to male input mRNA	855	772	Signaling	Synapse	Neuropeptide receptor binding	Pleckstrin homology domain	Thor. Gang (471)
			Synaptic transmission	Neuron part	Hormone activity	C2 domain	Brain (470)
			Neurotransmitter secretion	Axon	Nucleotide binding		Larval CNS (431)
Enriched in female TRAP relative to female input mRNA	447	408	Signaling	Synapse	Neuropeptide receptor binding	M1F4G-like domain	Thor. Gang (275)
			Synaptic transmission	Neuron part	Hormone activity		Brain (240)
			Neurotransmitter secretion	Axon	Nucleotide binding		Larval CNS (230)
Enriched in TRAP relative to input mRNA in both sexes	310	296	Signaling	Synapse	Neuropeptide receptor binding		Thor. Gang (183)
			Synaptic transmission	Neuron part	Hormone activity		Brain (177)
			Neurotransmitter secretion	Axon	G-coupled protein receptor binding		Head (167)
Uniquely enriched in male TRAP relative to male input mRNA	545	476	Signal transduction	Organelle		Pleckstrin homology domain	Brain (293)
			Signaling	Synapse		Oxysterol-binding protein	Thor. Gang (288)
			Cell communication	Neuron part			Larval CNS (268)
Uniquely enriched in female TRAP relative to female input mRNA	137	112					Larval CNS (67)
							Brain (63)
							Thor. Gang (62)
Sex-specific exon enrichment in TRAP relative to input mRNA		11					

a Gene Ontology (GO) terms and protein domain enrichments were among the most significant for each list.

b The three fly tissues that had the largest number of genes with significantly high expression in the FlyAtlas data set.

We performed visual inspection of plots showing count distributions along the gene models for the 11 genes predicted to have different transcript isoforms enriched in the male and female TRAP samples. The plots show quantitative differences in count abundance between the male and female exons in the TRAP samples, with the exon detected in both male and female TRAP samples. However, Hr51, Pkc53E, and CG42684 have exons that have mapped reads only in one sex (Figure S2), suggesting that there are sex-specific transcript isoforms in *fru P1*-expressing neurons. For example, Hr51 has three internal exons that have reads in the male TRAP, but not in the female TRAP samples; this coincides with our previous findings that Hr51 is regulated by Fru^M^ (Dalton *et al.* 2013). These data indicate that sexual dimorphism in the translatome in male and female *fru P1*-expressing neurons are due, at least in part, to different transcript isoform usage.

### Differences in the translatome of fru P1-expressing neurons between males and females

To identify all significant differences in mRNAs that are actively translated in *fru P1* neurons between males and females, we identified the sets of genes with exons that have significantly different RNA-seq abundance between the *fru P1*-expressing TRAP samples from males and females. There are 1210 genes that are male-biased in the TRAP samples, and 331 genes that are female-biased (Figure 3, Table 3, and Table S7). There are 16 genes with exons whose bias is dependent on the particular exon/isoform examined, giving further evidence for differences in transcript isoforms in the male and female TRAP samples (Figure 3D, Table 3, and Table S7). *fru* is one of the genes we detect as having differences in transcript isoforms in the TRAP samples, as expected (Table S7). There are 19 genes of the 1210 male-biased genes that are annotated with the GO term male courtship behavior (Table S5), including *dissatisfaction* (*dsf*), which has been previously been implicated in underlying male courtship behavior (Finley *et al.* 1998).

**Table 3:** Differences between male and female TRAP mRNA

Description	Exons	Genes	GO: Biological Process*^a^*	GO: Cellular Component*^a^*	GO: molecular function*^a^*	Protein Domain*^a^*	Tissue Expression (# of Genes)*^b^*
Male-biased TRAP mRNA	1420	1210	Signaling	Synapse	Ion binding	PDZ domain	Brain (732)
			Development	Cell junction	Cytoskeletal binding	SH3 domain	Larval CNS (723)
			Synaptic transmission	Presynapse		Pleckstrin homology domain	Thor. Gang (700)
Female-biased TRAP mRNA	415	331	Mitotic spindle	Ribosomal subunit	Structural constituent of ribosome		Adult carcass (182)
			translation				Adult heart (172)
							Head (162)
Sex-biased exon		16					

a Gene Ontology (GO) terms and protein domain enrichments were among the most significant for each list.

b The three fly tissues that had the largest number of genes with significantly high expression in the FlyAtlas data set.

Additionally, we determined how many genes are detected in TRAP samples from both males and females, and find 7967 genes (hereafter called core genes; Table S3). These core genes are significantly enriched with those that encode proteins with the Immunoglobulin-fold domain (163 genes; Table S5). We previously found genes encoding proteins with this domain were highly enriched in the gene sets identified as regulated by Fru^M^ (Goldman and Arbeitman 2007; Dalton *et al.* 2013). Furthermore, we found that one member, *dpr1*, is important for courtship gating (Goldman and Arbeitman 2007). These proteins have been shown to interact with each other, and to mediate specificity in synaptic connectivity (Ozkan *et al.* 2013; Carrillo *et al.* 2015; Tan *et al.* 2015).

*fru P1*-expressing neurons have a large set of core genes expressed in both males and females and a striking ∼4 times more genes with male-biased abundance than female-biased. We further examined the data to determine if there were any systematic biases in the male or female TRAP libraries that could account for detecting more male-biased genes. For all TRAP libraries the 3′ most exon is 37% of exons. If we remove the 3′ most exon from the analyses, this does not change the finding of dramatically increased male-biased TRAP mRNA levels.

### Examination of genes regulated by Fru^M^ and those with male-biased TRAP abundance

Of the 1210 genes that are significantly male-biased in the TRAP experiment, we investigated if these were potential targets of Fru^M^, by examining the overlap between the 1210 genes identified in this experiment to the 1253 genes with transcripts identified as being regulated by at least one of three Fru^M^ isoforms, from our previous RNA-seq study (Dalton *et al.* 2013). In Dalton *et al.* (2013), each of three Fru^M^ isoforms was overexpressed in *fru P1*-expressing neurons, and tissue was collected from 16- to 24-hr-old adults, to facilitate the identification of genes regulated by Fru^M^. Analysis of this data set was performed using the same mapping approaches to those used here, although the strain backgrounds are different. As this was an overexpression study, it is possible that Fru^M^ does not normally regulate some of the genes identified at the adult stage we examined. Furthermore, we do not expect every gene expressed in *fru P1*- expressing neurons to be a Fru^M^ target. We do find a significant overlap with 250 genes identified in the two studies (Table S7 and Table S8).

**Figure 4:**
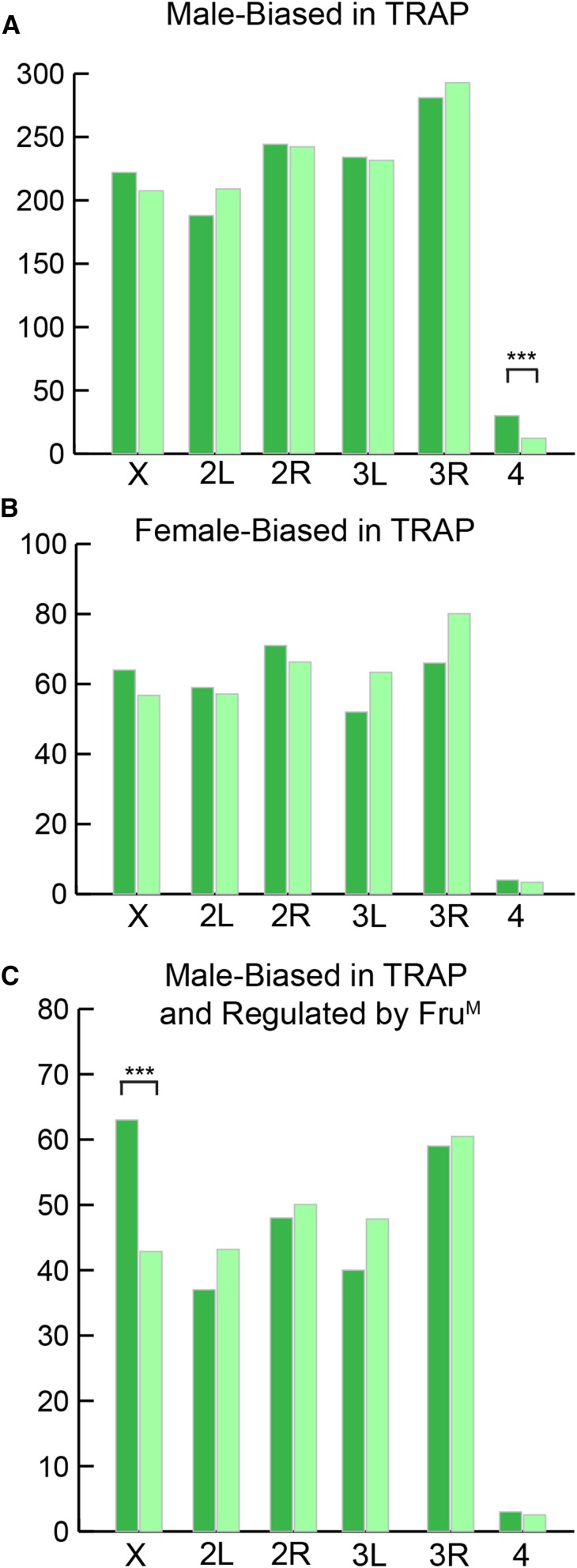
Chromosome distribution of genes with biased TRAP expression. Chromosome distribution of the (A) 1210 genes identified with male-biased TRAP expression, (B) 331 genes identified with female-biased TRAP expression, and (C) 250 genes identified with male-biased TRAP expression and overlap with previously identified Fru^M^ regulated genes (Dalton *et al.* 2013). The observed (dark green bars) and expected (light green bars) gene counts for each chromosome are shown. *** p < 0.001 using a Fisher’s exact test.

We also determined if there were significant differences in chromosome distribution for genes with male- and female-biased TRAP abundance, and found that genes with male-biased TRAP abundance were significantly enriched on the fourth chromosome; we found genes with sex-biased expression, and also those downstream of *dsx* highly significantly enriched on the fourth chromosome (Arbeitman *et al.* 2016). Furthermore, the 250 genes that have male-biased TRAP abundance, and were detected in our previous study as induced by Fru^M^, are significantly enriched on the X chromosome (Figure 4 and Table S8), as are the larger set of 1253 genes regulated by Fru^M^ (Dalton *et al.* 2013). To assess if there is evidence for direct regulation of these 250 genes, and the set of 1210 genes with male-biased TRAP abundance, we analyzed the frequency of each Fru^M^ DNA binding site motif in these genes, and found significant enrichment for the binding sites of three Fru^M^ isoforms with known DNA binding motifs (p < 0.0001; Table 4 and Table S8) (Dalton *et al.* 2013). This suggests that these sets of genes include many that are likely directly regulated by Fru^M.^

**Table 4:** Enrichment of Fru^M^ DNA binding motifs

		Total Genes	Number of Genes with Motif	Expected Number of Genes	Chi-Square Value	Degrees of Freedom	Likelihood Ratio Chi-Square	Fisher Raw p-Value (2-Tail)
Genes with male-biased TRAP mRNA*^a^*	Fru^A^ DBD Motif	1210	936	251.23	2782.216	1	2274.4412	< 0.0001
	Fru^B^ DBD Motif	1210	451	135.43	977.9614	1	731.1497	< 0.0001
	Fru^C^ DBD Motif	1210	273	85.432	523.3722	1	385.0885	< 0.0001
Genes with male-biased TRAP mRNA*^b^*	Fru^A^ DBD Motif	250	234	51.908	832.5475	1	651.4013	< 0.0001
and regulated by Fru^M^	Fru^B^ DBD Motif	250	137	27.982	493.9181	1	298.8676	< 0.0001
	Fru^C^ DBD Motif	250	94	17.651	366.9582	1	202.3083	< 0.0001

a Genes with male-biased TRAP mRNA relative to female TRAP mRNA (Table 3).

b Genes with male-biased TRAP mRNA relative to female TRAP mRNA (Table 3) and identified as regulated by Fru^M^ isoforms (Dalton *et al.* 2013).

## Discussion

We have used cell-type-specific TRAP to identify the repertoires of *Drosophila* genes with actively translated mRNA products in male and female *fru-P1*-expressing neurons from heads of young adult flies. This allows us to gain insight into how two vastly different behaviors are directed by a largely shared set of *fru P1*-expressing neurons and from a shared genome. We find 1210 genes have male-biased TRAP abundance, which is ∼4 times more than the 331 genes with female-biased TRAP abundance. This asymmetry in sex-bias is unique relative to many other studies examining sex-biased gene expression, in that similar numbers of genes were discovered to have male- or female-biased mRNA abundance in somatic tissues (Arbeitman *et al.* 2004; Goldman and Arbeitman 2007; Lebo *et al.* 2009; Chang *et al.* 2011), although, we did see a large asymmetry in sex-biased gene expression with more male-biased gene expression (331 male; six female) in the developing third larval instar genital disc, using microarray analyses (Masly *et al.* 2011).

Consistent with previous studies, the number of genes with sex-biased expression in the whole head (input here) is similar between the two sexes in this study. The 1210 male-biased genes in the *fru P1* mRNA translatome significantly overlap with genes that are regulated by Fru^M^ (250 genes overlap) (Dalton *et al.* 2013); the 250 genes common to both studies are also enriched on the X chromosome. Furthermore, the 1210 genes that have male-biased TRAP mRNA abundance are highly significantly enriched on the fourth chromosome; this is also true for genes with sex-biased expression and genes regulated by *dsx* in head tissues (Arbeitman *et al.* 2016). This is noteworthy, as the fourth chromosome in *D. melanogaster* evolved from an ancestral sex chromosome (Vicoso and Bachtrog 2013).

In our previous work examining head tissues, we found genes with male-biased expression are increased on the X chromosome and reside adjacent to dosage compensation machinery entry sites (Goldman and Arbeitman 2007; Chang *et al.* 2011), which was also observed separately (Catalan *et al.* 2012). Additionally, we found genes regulated by Fru^M^ to be enriched on the X chromosome (Dalton *et al.* 2013). We posited that this may be due to the unique properties of the X chromosome, which is less compacted and is also decorated with dosage compensation proteins in males. Fru^M^ function has evolved in the context of a male nucleus, and thus may have evolved biochemically to take advantage of the unique properties of the X chromosome in males. We further this idea, in light of our results presented here and previous work, by suggesting that the fourth chromosome also has unique properties for genes with sex-specific functions (Arbeitman *et al.* 2016).

There are several hypothesis about differences in the evolutionary properties of the X chromosome (reviewed in Vicoso and Charlesworth 2006), including the “faster-X” evolutionary hypothesis, which, in part, proposes that hemizygosity of the X chromosome in males could lead to more rapid adaptive fixation of male beneficial recessive alleles, as compared to autosomes (Rice 1984; Charlesworth *et al.* 1987). If targets of Fru^M^ are more likely to be on the X, the faster X may explain some of the male-bias in genes regulated by Fru^M^ identified here and previously. A gene’s residence on the X chromosome could allow for more rapid fixation of novel enhancer sequences that allow for Fru^M^ binding in males. Once these genes are under Fru^M^ regulation, selection could more rapidly act on X chromosome genes to retain new alleles/polymorphisms that are male beneficial, especially since regulation by Fru^M^ will have no impact in females.

We found that *fru P1* neurons from males and females have a common repertoire of enriched, actively translated mRNAs (core genes) that is distinct from those identified using TRAP to detect mRNAs from all neurons in the adult head (see Thomas *et al.* 2012). While *fru P1*-expressing neurons in males and females underlie very different complex reproductive behaviors, there are aspects of both behaviors that may utilize similar molecular processes, which might be unique to the *fru P1*-expressing class of neurons (reviewed in Greenspan and Ferveur 2000; Griffith and Ejima 2009; Ferveur 2010; Dauwalder 2011). For example, both male and female reproductive behaviors are plastic and can be altered by adult experiences. Furthermore, reproductive behaviors in males and females require social interactions, and are coordinated with nutritional status, germline status, and circadian rhythms. The results presented here suggest that there is a common set of core genes that have products in the actively translated pool of mRNAs that may underlie the abilities of the neurons to coordinate these functions in both males and females.

The asymmetry in the number of genes with higher expression in male TRAP samples as compared to females (∼4 times more male-biased genes), and the relatively small number of genes with female-biased expression (331), suggests that the potential for female behaviors directed by *fru P1*-expressing neurons may largely be specified by the core set of genes. Female reproductive behaviors include subsets of behaviors found in both sexes, like rejection with wing flicks and running away, but also include behavioral subsets that are uniquely female, such as oviposition. It is possible we did not assay the critical *fru P1*-expressing neuronal populations, where transcripts important for specifying female-specific neuronal architecture and physiology are translated, such as neurons in the ventral nerve cord. Furthermore, it is known that there are *fru P1* independent pathways that specify adult reproductive behaviors. There are Dsx^F^+ neurons that do not overlap with *fru P1*-expressing neurons that may direct aspects of female-specific behaviors (Lee *et al.* 2002; Sanders and Arbeitman 2008; Rideout *et al.* 2010; Robinett *et al.* 2010). Both *dsx* and *retained* function in females to repress male behaviors, whereas in males *dsx* promotes and *retained* represses male behaviors (Ditch *et al.* 2005; Shirangi *et al.* 2006). Also, in the absence of *fru P1*, males can learn to perform courtship behavior in a *dsx*-dependent manner (Pan and Baker 2014), demonstrating that there are additional mechanisms to direct the potential for courtship behavior in males. Additional studies examining more time points and cell types are important to fully understand how sex differences in behaviors are built in both males and females, especially during developmental pupal stages, which has been shown to be a critical time for the specification of sexually dimorphic behaviors (Belote and Baker 1987; Arthur *et al.* 1998). Furthermore, it will be important to understand the endogenous physiological differences in male and female *fru P1*-expressing neurons, in light of the observation that activation of *fru P1*-expressing neurons in females induced performance of a male courtship sub-behavior: unilateral wing extension/vibration, though the song produced is structured differently than wild type male song (Clyne and Miesenbock 2008).

The results presented here may also provide mechanistic clues as to how a shared genome and shared *fru P1*-expressing neuronal substrates can give rise to two distinct behavioral outcomes. It is possible that Fru^M^ functions to promote male-specific behavior in the context of the same gene regulatory networks that underlie female behavior. This may be because the genetic specification of male and female behaviors is constrained at the molecular level, as genes are present in both males and females and share regulatory elements. This does not mean that female behaviors are simply the default state, as that would suggest too large of a constraint on the specification and evolution of female behaviors. We suggest that complexity in function and evolution of reproductive traits in both males and females can arise using molecular pathways that are activated in both male and female neurons. This is possible because evolutionary constraints differ between males and females, given that regulation by Fru^M^ can decouple the impact of novel regulation between the sexes.

If most genes that underlie the potential for female behaviors are also expressed in males, in *fru P1*-expressing neurons, selection on new alleles that favorably alter female behavioral traits will occur in both sexes. The new beneficial mutations that impact female behaviors are likely to have some phenotypic effects in both sexes, since based on our translatome study here, they are likely to be expressed in both males and females. However, constraint on evolution of female behaviors may be reduced if the specification of male behaviors is robust enough that perturbation in the pathways that direct female-behaviors have a small impact on male behaviors. To direct robust male behaviors, many genes could be recruited to be regulated by Fru^M^, through addition of Fru^M^ binding sites, which will only have an impact in males. While there are elaborate male courtship displays, the evolution of the male behavioral process is constrained by female mate choice and the consequences on her fitness. Indeed, when male evolution continues in a context where female evolution is experimentally arrested, male fitness is increased and female fitness is decreased (Rice 1996,^1998^), given that selection on sexually antagonistic alleles has been removed.

Building a social behavior with a shared genome and a largely shared *fru P1*-expressing neuronal substrate may also limit the behavioral differences between males and females of a species. Thus, new alleles impacting sociality that benefit both males and females can be selected for in a shared genome. Further studies examining the evolution of Fru^M^ targets will inform on this question, especially focusing on X, fourth chromosome, and autosomal differences in evolution.

### Conclusions

*fru P1*-expressing neurons have a unique, sex-nonspecific TRAP profile, compared to the TRAP profile of all neurons of the adult head, suggesting that these shared molecular properties underlie common reproductive behavioral needs. This core set of TRAP genes are significantly enriched with those that encode immunoglobulin domain-containing superfamily members. The *fru P1*-expressing neurons control very different sex-specific reproductive behaviors. These data suggest that additional expression/translation of genes in males plays a large role in the differences in the functions of these neurons between males and females. This suggests a model for a rewriting of the neuronal program where a baseline pattern is laid, largely setting up the potential for female-specific behaviors, and then information is overlaid to create the potential for male-specific behaviors.

## Supplementary Material

Supplemental Material
