# Supplementary material for: Neurons That Underlie Drosophila melanogaster Reproductive Behaviors: Detection of a Large Male-Bias in Gene Expression in fruitless-Expressing Neurons
Source: G3 (Bethesda). 2016 May 31;6(8):2455–65. doi: 10.1534/g3.115.019265 (PMC4978899; doi:10.1534/g3.115.019265)
Supplement: Supplemental Material [file supp_6_8_2455__index.html]

Neurons That Underlie Drosophila melanogaster Reproductive Behaviors: Detection of a Large Male-Bias in Gene Expression in fruitless-Expressing Neurons — Supplemental Material 

# Neurons That Underlie *Drosophila melanogaster* Reproductive Behaviors: Detection of a Large Male-Bias in Gene Expression in *fruitless*-Expressing Neurons

## Supplemental Material for Newell *et al.*, 2016

**Files in this Data Supplement:**

- Figure S1 - Schematic description of exon level mapping. (.pdf, 342 KB)
- Figure S2 - Sequencing read coverage of genes that have sex specific exon usage. (.pdf, 536 KB)
- Table S1 - Full data set. (.xlsx, 24,466 KB)
- Table S2  - Number of differentially expressed genes using FDR p-value cut-off <0.2 and
- Table S3 - Detected Genes. (.xlsx, 6,240 KB)
- Table S4 - Differentially expressed genes between male and female input mRNA. (.xlsx, 602 KB)
- Table S5 - Gene Ontology Enrichment Results. (.xlsx, 4,691 KB)
- Table S6 - Genes significantly enriched in TRAP over input. (.xlsx, 1,793 KB)
- Table S7 - Differentially expressed genes between male and female TRAP mRNA. (.xlsx, 378 KB)
- Table S8 - Enrichment Tests. (.xlsx, 25 KB)
