# Supplementary figures and images for: Neurons That Underlie Drosophila melanogaster Reproductive Behaviors: Detection of a Large Male-Bias in Gene Expression in fruitless-Expressing Neurons
Source: G3 (Bethesda). 2016 May 31;6(8):2455–65. doi: 10.1534/g3.115.019265 (PMC4978899; doi:10.1534/g3.115.019265)

Figure S1

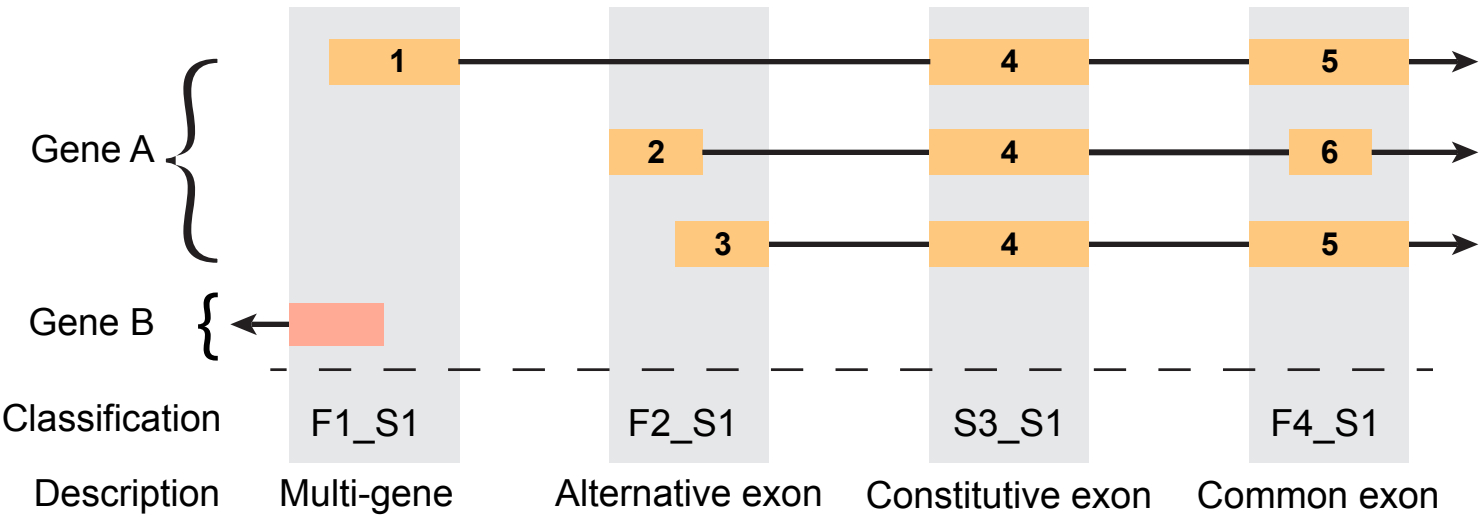

Supplement: Supplemental Material [file supp_g3.115.019265_FigureS1.pdf]
