# Supplementary material for: Neurons That Underlie Drosophila melanogaster Reproductive Behaviors: Detection of a Large Male-Bias in Gene Expression in fruitless-Expressing Neurons
Source: G3 (Bethesda). 2016 May 31;6(8):2455–65. doi: 10.1534/g3.115.019265 (PMC4978899; doi:10.1534/g3.115.019265)
Supplement: Supplemental Material [file supp_g3.115.019265_FigureS2.pdf]

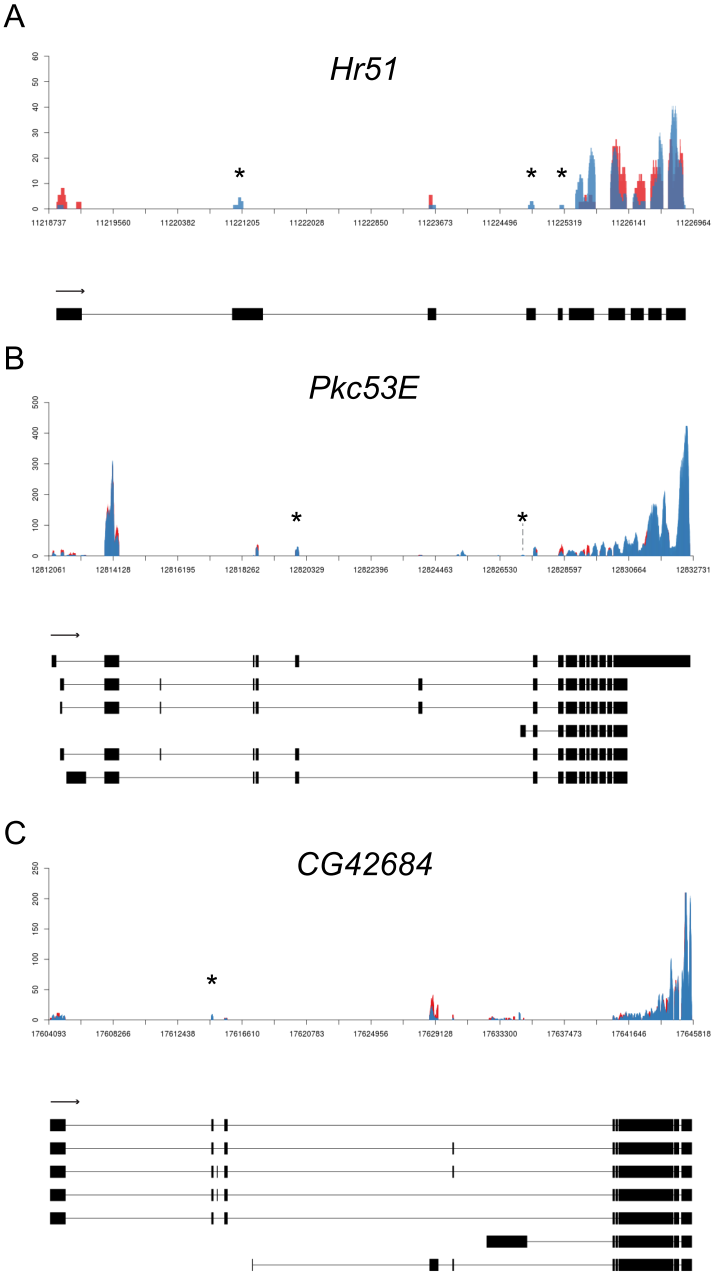

Supplemental Figure 2: Illustration of exon gene regions that have male-specific read counts

Gene models are shown for (A) *Hr51*, (B) *Pkc53E* and (C) *CG42684* with black boxes indicating exons and lines indicating intron regions. Exons that have read counts detected only in males are indicated by an asterisk (*). Above gene models is the RNA-seq mapped reads: blue is data from male TRAP samples and red is data from female TRAP samples. Arrow indicates the direction of transcription, with arrow over first exon.
